# Comparison of insertion torque, implant stability quotient and removal torque, in two different implant designs with and without osseodensification. - An ex vivo bench top study

**DOI:** 10.1016/j.jobcr.2023.02.004

**Published:** 2023-02-14

**Authors:** Yazad Gandhi, Ninad Padhye

**Affiliations:** a Consultant Maxillofacial Surgeon, Saifee Hospital, Mumbai, India; b Private Practice, Mumbai, India; c Clinical Research Fellow, Queen Mary University and the London School of Medicine and Dentistry, The Royal London Dental Hospital, London, UK

**Keywords:** Densah burs, Osseodensification, Primary stability, Thread design

## Abstract

**Background:**

Primary stability is an important factor in influencing the outcome of dental implants. Osteotomy modification techniques mentioned, include osteotomes for bone condensation, under-preparation of osteotomy and Osseodensification (OD). The objective of our twin arm study was to assess how two different implant designs respond to conventional osteotomy drilling and how these values obtained compare with OD.

**Materials and methods:**

The study comprised a total of 80 implants inserted in pig tibia bone. Group 1a (n = 20) consisted of tapered internal implants and group 1b (n = 20) consisted of tapered pro implants, both inserted with conventional drilling. Group 2a (n = 20) consisted of tapered internal implants and group 2b (n = 20) consisted of tapered pro implants, both inserted with OD. Each implant inserted was measured for implant stability quotient (ISQ), insertion torque and removal torque.

**Results:**

Group 1a showed a significantly lower ISQ, mean insertion and removal torque and as compared to Group 1b. Group 2a and 2b had comparable mean values for all the three parameters. Inter-group comparison showed a higher ISQ and insertion torque value for group 2 than group 1. Intra-group assessment showed a significantly lower value for all parameters for sub-group a than b.

**Conclusions:**

OD enhances primary stability of implants in bone; but when no OD is used, the tapered pro implant design offers a better primary stability. This may be attributed to the active thread design.

## Graphical abstract

**Figure:**
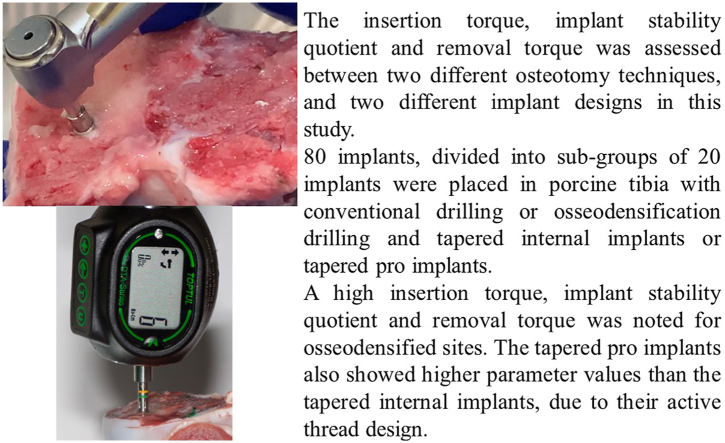

## 1 Introduction

Functional rehabilitation with dental implants depends on successful osseointegration, which in turn is influenced by the primary stability of an implant when inserted into bone. This primary stability depends on bone quantity, quality and implant macro-design alongside the osteotomy protocol used. Secondary stability on the other hand is influenced by the biologic bond between titanium and bone by way of osseointegration.^1^

Conventional drilling protocol, often referred to as extraction drilling, involves the use of system specific rotary drills in clockwise rotation to sequentially extract bone from within the osteotomy. This sometimes reduces the primary stability of the implant inserted, especially when working with soft bone (type D3 or D4).^2^^,^^3^ Several methods and devices have been used to evaluate primary stability of implants.^4^^,^^5^ But among these, the resonance frequency analysis which measures the implant stability quotient (ISQ), has shown to be the most reliable method.^6^ Technique specific factors include under-prepared osteotomies,^7^^,^^8^ bone condensation using osteotomes,^9^^,^^10^ and a more recently described Osseodensification technique.^11^ Almutairi et al. compared 4 different thread designs with insignificant differences in Periotest values when inserted using conventional drilling versus Osseodensification (OD).^7^

In the protocol for Osseodensification, specially designed burs work in counterclockwise motion to produce lateral and apical cancellous compaction thereby increasing immediate bone-implant contact. This significantly enhances the primary stability of the implant thereby facilitating immediate provisionalization.^11^, ^12^, ^13^

In this study, we compared two different implants with a similar body geometry but different thread design for primary stability, when inserted using the conventional drilling protocol vis a vis the Osseodensification protocol. The Biohorizons® tapered pro implant system has a progressive 6 mm apical taper, similar to the tapered internal implants, but is set with threads that are 33% deeper. This arguably lends higher primary stability to implants inserted in soft bone.^14^

Rationale of this twin arm study was: (A) To infer any benefit of the more aggressive thread design in the Biohorizons Tapered Pro® implants compared to the older Biohorizons® Tapered internal system, when inserted using the manufacturer's conventional drilling protocol and (B) Whether Osseodensification protocol improves the primary stability for this deeper thread design. To the best of our knowledge there is no known preclinical research to compare Biohorizons® tapered implant systems with the Tapered Pro system with respect to insertion torque, removal torque and ISQ. This study may provide critical information by quantifying the effect of thread depth and design on insertion torque and ISQ, thereby lending vital information for a possible randomized clinical trial comparing such implant designs.

## 2 Materials and Methods

An ex vivo experimental bench top study was designed in accordance with the ethical standards outline in the 1964 Declaration of Helsinki, as revised in 2008. Commercially available cuts of porcine tibia were procured the same day of animal death. No animals were sacrificed for this study and all bone samples procured were those which were discarded at the local abattoir. The bone was prepared by removing all attached soft tissue and exposing a flat cancellous table.

In this twin arm study, an arbitrary sample size of 80 osteotomies was selected and divided into 2 groups depending on implant type used and further divided into 2 subgroups depending on the osteotomy method.

Group 1a: Biohorizons® tapered internal implant inserted using conventional drilling.
Group 1b: Biohorizons® tapered pro implant inserted using conventional drilling.
Group 2a: Biohorizons® tapered internal implant inserted using Osseodensification drilling.
Group 2b: Biohorizons® tapered pro implant inserted using Osseodensification drilling.

All bone samples were mounted on a press so that they were stable during drilling. All samples were marked A,P,M,L corresponding with anterior, posterior, medial and lateral surfaces for future correlation. A surgical motor with speed and torque control (Aseptico 7000E series, Aseptico Inc, Washington, USA) was used with copious amount of chilled saline irrigation. Maximum available torque on the motor was 80Ncm. All osteotomies were planned with an inter-osteotomy distance of 10 mm.

**Fig. 1:**
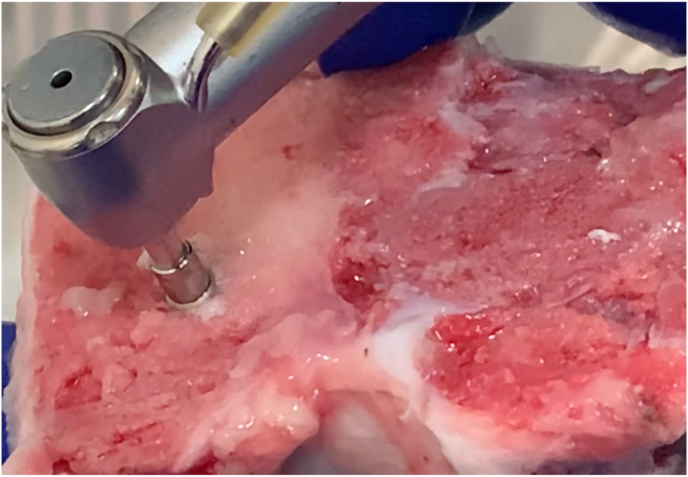
Osteotomy and implant placement in porcine tibia.

In Group 1a and 1b, the osteotomies were performed as per the manufacturer's recommendation using the (Biohorizons® Tapered HD surgical system, Biohorizons®, Birmingham, Alabama, USA) starting with the 2.0 mm pilot drill, advancing onto the 2.5 mm twist drill and the final 3.2 mm width drill. All drills advanced to a depth of 12 mm at 1200 rpm and 30Ncm torque under copious saline irrigation (Fig. 1).^15^

In Group 2a and 2b the osteotomies were prepared using Densah drills (Versah®, Jackson, Michigan, USA) in a 3-drill sequence-a 2.0 mm pilot drill used clockwise at 1200 rpm, followed by VT1525 drill and finally the VT2535 in counter-clockwise rotation at 800 rpm under copious saline irrigation. All drills advanced to a depth of 12 mm.^16^

Biohorizons® tapered internal implants 3.8 × 12mm were inserted in Groups 1a and 2a while Groups 1b and 2b received Biohorizons® tapered pro implants 3.8 × 12 mm using the handpiece and graduated incremental torque delivered via the implant motor. The motor had an auto-setting of 15 RPM for implant insertion, with the torque adjusted at 20Ncm. This would gradually increase after each stall of the handpiece, until each implant was at the bone level. Motor torque at final implant position was noted.

**Fig. 2:**
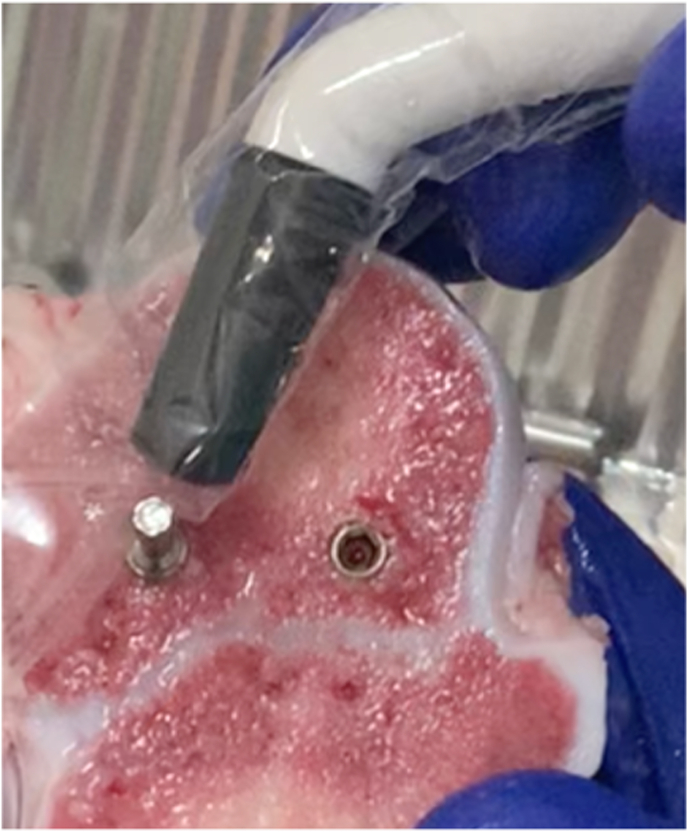
Measurement of implant stability quotient (ISQ) for the inserted implants.

ISQ values were measured using the Penguin RFA instrument (Penguin, Integration Diagnostics, Göteborg, Sweden) from 4 different directions anterior, posterior, medial, lateral and an average value obtained for each insertion (Fig. 2).

**Fig. 3:**
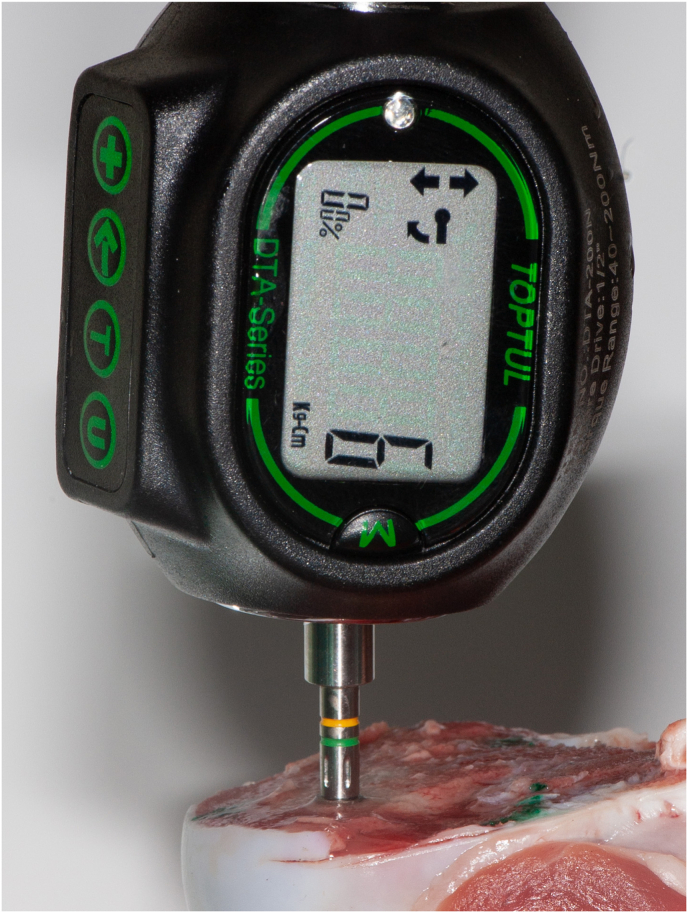
Measurement of the implant removal torque.

Following these measurements, the implant was removed using the implant motor and handpiece. The torque setting for removal was initially set at 10Ncm lower than the final insertion torque of the respective implant. The torque was gradually increased till the implant started rotating in reverse and this value was noted (Fig. 3).

## 3 Statistical analysis

The data was analysed using SPSS 21.0 software. Values have been depicted as mean ± standard deviation. ANOVA followed by Tukey HSD test (post-hoc) have been used to compare the data. ‘p’ value less than 0.05 was considered as significant (95% confidence interval).

## 4 Result

A total of 80 osteotomy sites in porcine tibia were assessed in this ex vivo bench top study. The osteotomy sites were divided equally between the groups, thus each sub-group had a total of 20 implants. All the implants were placed by a single operator, to eliminate inter-operator discrepancy.

The mean insertion torque for groups 1a, 2a, 1b and 2b were 34.5 ± 3.59 Ncm, 46.5 ± 3.28 Ncm, 43.5 ± 2.86 Ncm and 46.5 ± 32.8 Ncm respectively. Inter-group showed a significantly higher insertion torque (*p* < 0.001) for groups 2a and 2b. However, no significant difference was noted between groups 2a and 1b (*p* = 0.024). The ISQ ranged from 57 to 79 for all the groups. Groups 2a and 2b had the highest ISQ value at 75.85 ± 2.32, which was significantly higher than group 1a (59.85 ± 1.42) and 1b (71.9 ± 2.40) (*p* < 0.001). Highest mean removal torque (47.25 Ncm) was noted for groups 2a and 2b, while the lowest torque was seen for group 1a. The removal torque was highest for groups 2a and 2b, followed by 1b and then 1a (Table 1, Table 2).

**Table 1:** Comparison of mean insertion torque with densification, implant stability quotient (ISQ) and removal torque with densification among different groups.

	Group	N	Mean	Std. Deviation	Minimum	Maximum	F (ANOVA)	‘p’
Insertion torque (Ncm)	1a	20	34.50	3.59	30.00	40.00	60.52	<0.001
	2a	20	46.50	3.28	40.00	50.00		
	1b	20	43.50	2.96	40.00	50.00		
	2b	20	46.50	3.28	40.00	50.00		
Implant stability quotient (ISQ)	1a	20	59.85	1.42	57.00	62.00	246.71	<0.001
	2a	20	75.85	2.32	70.00	79.00		
	1b	20	71.90	2.40	68.00	76.00		
	2b	20	75.85	2.32	70.00	79.00		
Removal torque (Ncm)	1a	20	35.50	3.94	30.00	45.00	61.22	<0.001
	2a	20	47.25	3.02	45.00	55.00		
	1b	20	43.00	2.51	40.00	45.00		
	2b	20	47.25	3.02	45.00	55.00

**Table 2:** Inter-group comparison of mean Insertion torque, Implant stability quotient and Removal torque (Tukey HSD test).

SN	Comparison	Insertion torque (Ncm)			Implant stability Quotient (ISQ)			Removal torque (Ncm)		
		Mean Diff.	SE	‘p’	Mean Diff.	SE	‘p’	Mean Diff.	SE	‘p’
1.	1a vs 2a	−12.00	1.03	<0.001	−16.00	0.68	0.000	−11.75	1.00	0.000
2.	1a vs 1b	−9.00	1.03	<0.001	−12.05	0.68	0.000	−7.50	1.00	0.000
3.	1a vs 2b	−12.00	1.03	<0.001	−16.00	0.68	0.000	−11.75	1.00	0.000
4.	2a vs 1b	3.00	1.03	0.024	3.95	0.68	0.000	4.25	1.00	0.000
5.	2a vs 2b	0.00	1.03	1.000	0.00	0.68	1.000	0.00	1.00	1.000
6.	1b vs 2b	−3.00	1.03	0.024	−3.95	0.68	0.000	−4.25	1.00	0.000
Order		2a = 2b > 1b > 1a			2a = 2b > 1b > 1a			2a = 2b > 1b > 1a		

Group 2a and 2b had comparable mean values for all the three parameters. Both the groups had significantly higher mean value as compared to Groups 1a and 1b. Between Groups 1a and 1b Group 1a had significantly lower mean values as compared to Group 1b.

## 5 Discussion

It is not uncommon to find clinical situations with low cancellous bone density in aging population or even young individuals, especially in the posterior maxilla. Implant thread designs with varying shapes are available in the market ranging from ‘V’ to ‘Square’ to ‘Buttress’ threads. These offer different levels of primary stability depending on the lateral compressive forces on bone.^2^ When Almutairi et al. compared 4 different thread designs for differences in Periotest values, they argued that increasing thread depth and decreasing thread pitch could lead to higher primary stability in soft bone.^7^

Osteotome compaction has been used since more than two decades to increase the bone density at the insertion site with varying results.^9^ However, as a drawback, uncontrolled application of condensing force with the use of osteotomes may produce higher bone density without significantly affecting primary implant stability and hinder secondary stability by affecting osseointegration due to micro fractures. OD on the other hand produces a more controlled environment of cancellous autograft in the immediate vicinity of the implant. Clinical research shows that implants with higher primary stability have a better survival rate (≥96.9%).^10^^,^^11^ In our study, the OD groups showed a significantly higher insertion torque, ISQ and removal torque as compared to the conventional drilling group, for both the types of implants used. The insertion torque, ISQ and removal torque were similar for groups 2a and 2b (implants placed using OD). This would suggest that OD osteotomy technique increases the primary stability, irrespective of the thread design of the implants. However, when OD is not possible, then implants with an active thread design may be considered to increase the primary stability.

Huwais et al. used OD in a similar ex vivo study design with 72 parallel walled implants and showed a significant increment in insertion torque, removal torque and ISQ values when using Densah® drills in counter clockwise rotation.^11^ OD can produce a gradual and controlled expansion of the cancellous component of bone by lateral micro-compaction of bone thereby maintaining the osteogenic potential of the peri-implant bone.

Wang et al. have shown in their animal model that bone condensation using osteotomes does not significantly influence the implant primary stability in bone even though it increases the bone density.^12^ Higher primary stability helps achieve a more predictable secondary stability and facilitates early restoration or even immediate loading in certain situations.

A possible confounder in our study design may have been the diameter of implants used, had a larger diameter been used the difference in values may have been significantly higher. Lopez, Trisi and Huwais have compared conventional osteotomies and OD using different implant diameters which possibly led to differences in results but all reported a higher ISQ value using OD.^11^^,^^17^^,^^18^

Our study model using porcine tibia with major cancellous component and minimal cortical bone resembles human bone of Type III or IV found mostly in the posterior maxilla and mandible. A possible limitation of the study was the absence of follow-up and subsequent assessment of osseointegration. However, such ex vivo studies have the strength of providing higher number of observational data, which adds significantly to the literature. We stand in agreement that further studies by way of multi-centre randomized clinical trials comparing the Biohorizons® Tapered Internal and Tapered Pro implant systems would give valuable insight on this topic and the same is recommended.

## 6 Conclusions

Osseodensification is a proven method to augment primary stability of implants in bone. But when no osseodensification is used, Biohorizons Tapered Pro implants offer better primary stability when compared with the Tapered Internal implant design. This may be due to the difference in thread design and depth which may prove to be beneficial when implants are inserted in soft bone.

## Funding received

This research did not receive any specific grant from funding agencies in the public, commercial, or not-for-profit sectors.

## Declaration of competing interest

None.
